# Editorial: Cancer Cell Mechanobiology - A New Frontier for Cancer Invasion and Metastasis Research

**DOI:** 10.3389/fcell.2021.775012

**Published:** 2021-10-07

**Authors:** Jianyu Rao, Chwee Teck Lim, Tony Hu, Dan Han

**Affiliations:** ^1^Department of Pathology and Laboratory Medicine, University of California at Los Angeles, Los Angeles, CA, United States; ^2^Department of Biomedical Engineering, National University of Singapore, Singapore, Singapore; ^3^Department of Mechanical Engineering, National University of Singapore, Singapore, Singapore; ^4^Department of Biomedical Engineering, School of Medicine, Tulane University, New Orleans, LA, United States; ^5^Laboratory for Biological Effects of Nanomaterials and Nanosafety, National Centre for Nanoscience and Technology, Beijing, China

**Keywords:** cancer, cell, mechanobiology, mechanical property, invasion, metastasis

The phrase typically used to describe the physical characteristics of a cancer is “hard as a rock,” since a cancerous mass typically feels like rock that is fixed and hard to move around. Indeed, before the age of imaging and pathological diagnostic modalities, people often relied on physically touching a mass to determine if it was malignant.

Fast forwarding to twenty-first century, the recognition and analysis of cancer has evolved to encompass imaging-based diagnostic evaluations, including *in vivo* imaging (X-rays, CT, and MRI) and microscopic examination; immunohistochemical, and protein phenotype characterizations; and more recently to DNA/RNA molecular profiling analyses, including next generation sequencing (NGS). Thus, if one considers morphology as the 1st dimension, protein phenotypes as the 2nd dimension, and DNA/RNA molecular profiling as the 3rd dimension of cancer, how should we account for the physical properties of cancer and cancer cells as a 4th dimension to better understand cancer behavior?

Ultimately, it is usually the physical changes in a cancer that cause pathologies that eventually lead to death. These physical changes include but are not limited to uncontrolled growth of the cancer mass that compromises vital organ and tissue functions, *via* tissue disruption or compression effects; as well as cancer cell invasions into surrounding tissues and metastasis to other more distant sites to disrupt the function of affected or adjacent tissues by similar mechanisms. Thus, physical and mechanical properties of cancers and cancer cells can be viewed as a 4th dimension that directly correlate with their malignant nature.

Analysis of this 4th dimension is possible due to research advances on distinct fronts: (1) cellular and molecular biological research that has improved our understanding of molecular events leading to altered signaling pathways associated with cellular adhesion and cytoskeletal remodeling; and (2) the ability to measure cancer cell mechanical features using nanotechnological and microtechnological platforms. One first landmark study that employed Atomic Force Microscopy (AFM) to examine cancer cells found that metastatic cancer cells obtained from body fluid sample of patients were much “softer” than morphologically similar non-cancerous mesothelial cells (Cross et al., 2007). This observation has been repeatedly validated with various microfluidic platforms, including Deformability Cytometry (DC) and Permeability Filtration (PMF), by us and others (Gossett et al., 2012; Qi et al., 2015). This finding appears paradoxical to the notion that cancers are hard, yet is now explainable based on our understanding of cytoskeletal remodeling and the relationship of cancer cell-stroma interactions. While cancer cells, particularly invasive cancer cells, have altered adhesion properties and cytoskeletal structures that make them more flexible and hence “softer,” the stiff nature of the overall mass is the result of the stromal response (e.g., fibrosis and desmoplastic reactions) to the invasive cancer cells (Roy Choudhury et al., 2019).

A new research field—“cancer cell mechanobiology”—has emerged on the basis of these studies (Ladoux and Mège, 2017; Chaudhuri et al., 2018; Roy Choudhury et al., 2019). Subsequent studies further in this area have shown that the mechanical properties of analyzed cells can provide a quantitative assessment of their invasive and metastatic potential, which can be used as a diagnostic marker of metastatic cancer using samples derived from various body fluids and the tumor microenvironment. More importantly, these analyses may not only help us to identify and diagnose cancer cells more accurately, but may also more precisely determine the nature, or aggressiveness, of malignant cells. For example, there are currently no reliable morphological and molecular markers that can be used to determine whether a cancer cell identified in a patient's urine or fine needle aspirate specimen is invasive or not. This determination is clinically important as it distinguishes pre-invasive *in situ* cancers from invasive cancers that require more aggressive treatment. Further, one can envision not only utilizing cancer mechanobiological properties to screen for drug targets but also changing the cancer mechanics that directly inhibit cancer cell invasion and metastasis.

In view of recent progress in the field of cancer cell mechanobiology, we believe that it is an opportune time for a special edition dedicated to this topic and its implications for cancer evaluation. In this edition, we have compiled reviews and original research articles that cover key topics in this emerging area, ones that address molecular mechanisms underlying cancer cell and stroma mechanobiology, cancer cell mechanobiological studies in pre- and early malignant processes, and cancer cell mechanophenotype(s) as biomarkers for cancer detection or therapeutic monitoring. It is our sincere hope that this special issue will open a new research frontier that will eventually lead to improved approach to control and conquer this deadly disease.

## Author Contributions

All authors listed have made a substantial, direct and intellectual contribution to the work, and approved it for publication.

## Funding

This study was supported by NCI R21 CA208196 and JCCF Impact Grant.

## Conflict of Interest

The authors declare that the research was conducted in the absence of any commercial or financial relationships that could be construed as a potential conflict of interest.

## Publisher's Note

All claims expressed in this article are solely those of the authors and do not necessarily represent those of their affiliated organizations, or those of the publisher, the editors and the reviewers. Any product that may be evaluated in this article, or claim that may be made by its manufacturer, is not guaranteed or endorsed by the publisher.
